# First Case of Combined Carpal and Cubital Tunnel Syndromes in an Adolescent: A Case Report and Literature Review

**DOI:** 10.7759/cureus.65650

**Published:** 2024-07-29

**Authors:** Samantha N Olson, Thaddeus D Harbaugh, Mason T Stoltzfus, Sonia S Majid, Oliver D Mrowczynski

**Affiliations:** 1 Neurosurgery, Penn State Health Milton S. Hershey Medical Center, Hershey, USA

**Keywords:** ulnar nerve neuropathy, median neuropathy, pediatric, cubital tunnel, carpal tunnel syndome, peripheral nerve disorders

## Abstract

Carpal and cubital tunnel syndromes are the two most common compressive neuropathies, but both carpal and cubital tunnel syndromes are extremely rare in children. Therefore, the combination of carpal and cubital tunnel syndrome in the pediatric population is even more uncommon. These neuropathies have multiple causes, with the three main categories being mechanical injury, metabolic, and idiopathic. Here, we present the unique case of a 15-year-old female with no known genetic or physical risk factors who was diagnosed with atraumatic combined carpal and cubital tunnel syndrome with severe, chronic nerve entrapment and damage. After nearly two years of conservative management, the patient had a cubital tunnel and carpal tunnel release simultaneously. The transverse carpal ligament was grossly thickened intraoperatively, leading to difficulty in the identification of the median nerve. The ulnar nerve was severely compressed and flattened. Following decompression, both nerves continued to be erythematous and inflamed. After surgery, the patient had barriers to getting appropriate postoperative care. Specifically, the patient was unable to attend physical and hand therapy appointments, possibly leading to continued weakness, numbness, and intermittent pain. In our patient, the preoperative workup did not illuminate the severity of the median and ulnar nerve damage, possibly delaying surgical intervention. In addition to our case, we utilized the TriNetX database (TriNetX, Inc., Cambridge, Massachusetts, United States) to investigate the rate and treatment of compressive neuropathies in the pediatric population. The database was queried for pediatric patients who underwent carpal tunnel release, cubital tunnel release, and pediatric patients with both carpal and cubital tunnel syndrome diagnoses in childhood. We found that there were 20,819,207 pediatric patients on the TriNetX database, of whom 503 (0.002%) were diagnosed with both carpal and cubital tunnel syndrome. Based on our case and the current literature, a thorough history of pediatric patients with suspected carpal or cubital tunnel syndrome should include an evaluation of family history and activity level for pertinent risk factors. Widening the scope of the patient history could allow for more timely surgical intervention and improve long-term outcomes for the pediatric population. When evaluating children for either carpal tunnel or cubital tunnel syndrome, we recommend that healthcare providers evaluate both neuropathies simultaneously.

## Introduction

Carpal and cubital tunnel syndromes, caused by median and ulnar nerve compression, respectively, are the two most common compressive neuropathies [1-3]. In children, carpal tunnel syndrome is extremely rare and accounts for only 0.2% of all cases [4]. Ulnar mononeuropathy is children’s most common upper extremity mononeuropathy but is still considered rare in comparison to the adult cohort [5,6]. Consequently, the incidence of combined carpal and cubital tunnels in the pediatric population is exceptionally unusual. Ulnar neuropathy is most commonly a result of trauma but is also associated with cubitus varus deformity, the syndrome of hereditary neuropathy with liability to pressure palsies (HNPP), throwing activities, or anomalous anatomy [5,6]. There are many causes for these neuropathies. Some studies suggest that up to half of carpal tunnel syndrome cases occur secondary to lysosomal storage diseases and their hypertrophic effects on the transverse carpal ligament, tendons, and tenosynovium [5]. These diseases also lead to a delay in identification and treatment due to the coinciding mental disability [5]. Other etiologies of pediatric carpal tunnel syndrome include metabolic, mechanical, and idiopathic causes such as congenital bone and muscle anomalies, HNPP, growth hormone, hypothyroidism, Down syndrome, hemophilia, Schwartz-Jampel syndrome, scleroderma, physical activity, and others [5,6]. Pediatric carpal tunnel syndrome can manifest with or without the classic numbness and tingling of the first through third digits, nighttime pain, and paresthesia often experienced by adults. Pediatric carpal tunnel syndrome may also present with atrophy or weakness of the thenar muscles, clumsiness of the affected hand, and pain, making an accurate diagnosis difficult [5-7]. Tinel’s sign (tapping on the wrist of the patient to elicit signs of neuropathy) and the Phalen maneuver (hand maneuvers to elicit signs of neuropathy) are also often normal in children with long-standing median nerve compression, further complicating the diagnosis [5-7].

## Case presentation

A 15-year-old right-handed female presented with right upper extremity pain, swelling, paresthesia, and weakness that had persisted for 19 months. Before presenting at our medical center, she had been seeing rehabilitation physicians and working with pediatric physical medicine and rehabilitation doctors. Previous electromyography (EMG) and MRI studies performed 19 months prior showed mild ulnar neuropathy. MRIs of the brain and cervical spine were unremarkable. She had trialed various medications, including Tylenol, ibuprofen, a lidocaine patch, and gabapentin, as part of conservative management strategies. Recently, her pain had worsened, prompting her presentation at our clinic. She reported that the pain was worse at night and severe enough to interrupt her sleep. Additionally, she had difficulty picking up pencils at school, along with weakness and numbness with arm movement.

On physical examination, her pinprick sensation was diminished from her shoulder down to both the ventral and dorsal aspects of her right limb. Wrist flexion was weak at 4-, her hand intrinsics exhibited diminished power, and her radial deep and superficial flexors were weak at 4- on the ulnar side. The pinprick sensation was also diminished, with strength at 4- for the abductors of her hand and 4- for the adductor pollicis. Her family history was notable for her mother having carpal tunnel syndrome, but she was otherwise negative for any additional family history of nerve disorders.

**Figure 1:**
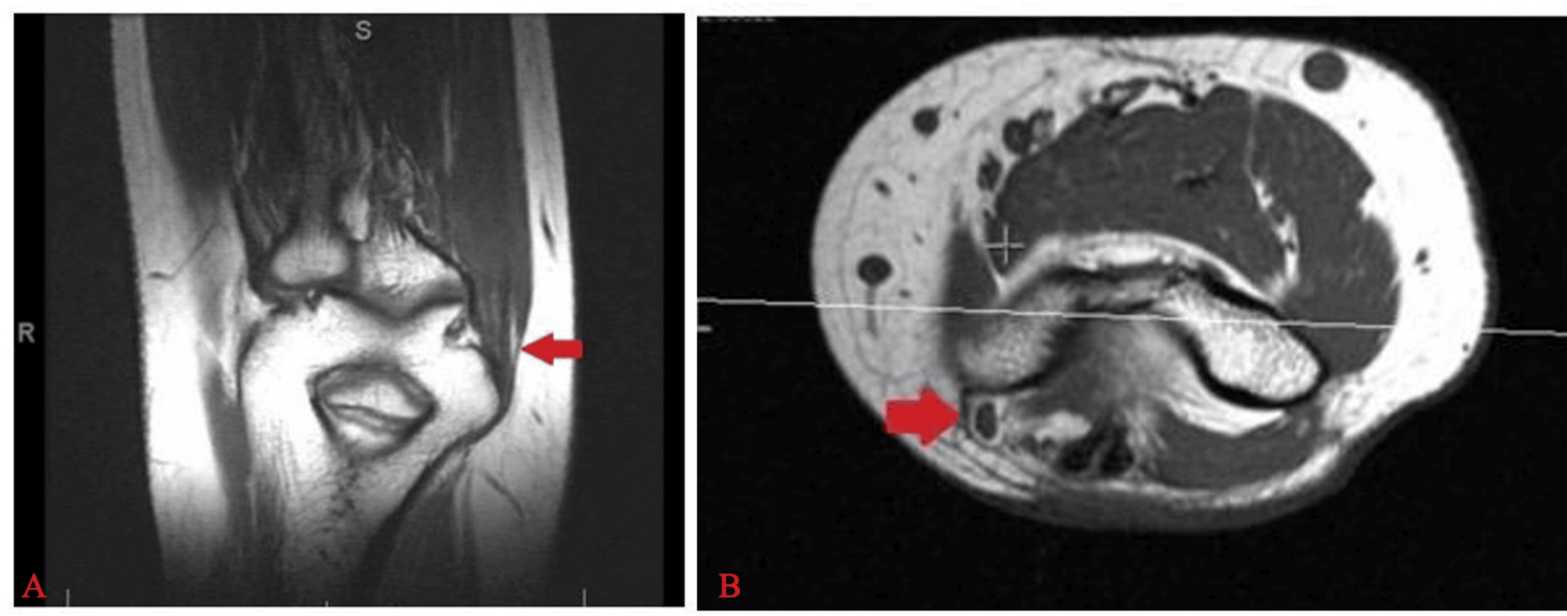
Preoperative ulnar nerve inflammation (A) Coronal T1-weighted MRI of the ulnar nerve, with the red arrow indicating ulnar nerve inflammation. (B) Axial T1-weighted MRI of the ulnar nerve, with the red arrow indicating ulnar nerve inflammation.

A right upper extremity EMG demonstrated evidence of chronic, mild median, and ulnar neuropathies. An MRI of the right upper extremity showed ulnar nerve edema and thickening in the cubital tunnel (Figure 1). A repeat EMG at the time of presentation to our clinic revealed signs of acute denervation of the median nerve and chronic radiculopathy affecting the C8-T1 nerve roots, along with changes in the proximal flexor pollicis longus. A subsequent cervical spine MRI was obtained to rule out more proximal neuropathy and showed no evidence of cervical nerve root entrapment or neuropathy. No nerve conduction studies were performed on this patient. The worsening EMG findings and progression of symptoms prompted the decision to perform combined carpal and cubital tunnel releases three months after her presentation to our clinic.

The patient underwent a high-frequency (MHz) B-mode, nonvascular ultrasound of her bilateral upper extremities. The ultrasound revealed evidence of entrapment of her right median and ulnar nerves. However, neither nerve met the established ultrasonographic criteria for diagnosing carpal and cubital tunnel syndromes, which are a cross-sectional area (CSA) greater than 12 mm² for the median nerve and 10 mm² for the ulnar nerve. The CSA of her right median nerve was 6.9 mm² in the palm and distal carpal tunnel and 8.8 mm² at the wrist and proximal carpal tunnel. The CSA of her right ulnar nerve was 5.2 mm² in the cubital tunnel, 7.9 mm² in the ulnar groove and medial epicondyle, and 5.6 mm² in the region proximal to the medial epicondyle.

**Figure 2:**
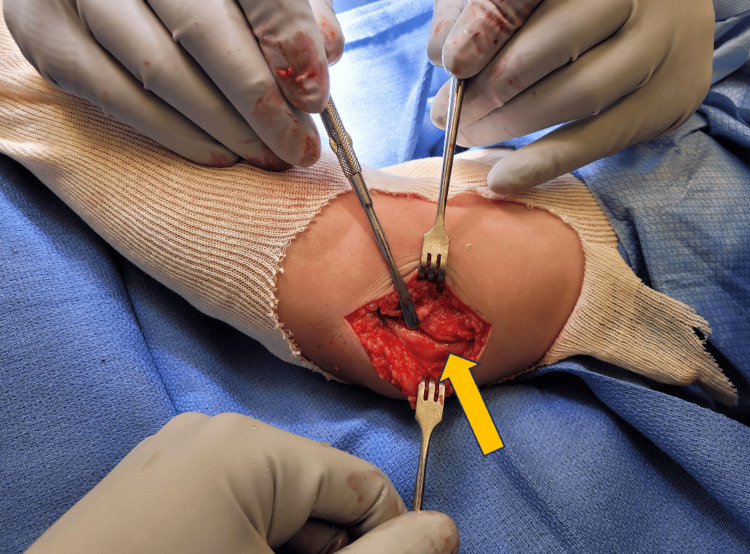
Right ulnar nerve (yellow arrow) following decompression proximally and distally

Intraoperatively, the transverse carpal ligament was observed to be grossly thickened, compressing the underlying median nerve to a degree that made it difficult to identify the nerve. The ulnar nerve was compressed and flattened by the regional aponeurosis and flexor carpi ulnaris fascia, and it was ultimately decompressed proximally and distally (Figure 2). The nerves appeared erythematous and inflamed upon full visualization following decompression. There were no intraoperative complications.

At the two-week postoperative follow-up, the patient was recovering well with an appropriately healed incision. She had worn her ace bandage from the day of the procedure, and her sutures had been removed. Postoperatively, she was advised to wiggle the fingers of her affected limb and was able to resume activity one day following the procedure. On physical examination, she exhibited minimal improvement in weakness, with continued issues in finger flexion, wrist flexion, wrist extension, and decreased sensation in all digits of the right hand. She was referred to hand therapy at this appointment.

At her seven-week follow-up, the patient was recovering from surgery appropriately but continued to experience weakness in the right upper extremity. On examination, she had 4-/5 strength in her biceps, triceps, wrist flexors, and wrist extensors, along with decreased grip strength in her right arm. She continued to have pain around the cubital tunnel incision and along the ulnar distribution of her right hand. She had been taking 300 mg of gabapentin orally three times daily with minimal relief.

Around four months following surgery, the patient complained of right-hand weakness, numbness, and intermittent pain without any improvement. She had attended only one hand therapy appointment a week before this four-month follow-up and had not participated in upper arm therapy. The patient reported that right arm swelling had occurred around three months after surgery, causing her to miss one week of school. During the period of swelling, she was examined and had an X-ray completed, but no fractures or infections were found. She had experienced no other issues with her arm since this incident and continued to take gabapentin three times daily. Her physical examination at the four-month appointment remained unchanged from previous exams. The patient continued to follow up in our clinic, and we were awaiting new EMG results. She had also been referred to neurology for further evaluation.

## Discussion

Combined cases of pediatric carpal and cubital tunnel syndrome are exceptionally rare, making it important to report such cases. To date, there are around 1,000 carpal tunnel and around 3,000 cubital tunnel cases in pediatric patients, as discussed on PubMed, but no cases of combined pediatric carpal and cubital tunnel syndrome have been reported. The existing literature finds that cubital tunnel syndrome can result from mechanical, idiopathic/atraumatic, and genetic conditions (Table 1).

**Table 1:** Causes of pediatric cubital tunnel syndrome HNPP, hereditary neuropathy with liability to pressure palsies Sources: Costales et al. (2019) [5]; Gallone et al. (2020) [6]

Mechanical	Idiopathic/atraumatic	Genetic conditions
Cubitus varus deformity	Throwing activities	Syndrome of HNPP
Fracture	Bike riding	Congenital constriction band syndrome
Laceration	Resting on wheelchair armrests	
Puncture wound	Hockey goalie equipment	
	Anomalous anatomy producing entrapment	

Carpal tunnel syndrome in the pediatric population can be related to metabolic, mechanical, and idiopathic causes (Table 2). When evaluating children with signs of cubital and/or carpal tunnel syndrome, inquiring about specific risk-factor activities and exploring their genetic background for evidence of HNPP and lysosomal storage diseases could help determine a diagnosis of ulnar and/or median nerve neuropathy. In patients without known risks, exploring other possible causative factors of severe medial and ulnar neuropathy is important.

**Table 2:** Causes of pediatric carpal tunnel syndrome * Most cases are attributed to genetic conditions ** Lysosomal storage diseases HNPP, hereditary neuropathy with liability to pressure palsies; ML, mucolipidosis; MPS, mucopolysaccharidosis Sources: Costales et al. (2019) [5]; Gallone et al. (2020) [6]

Metabolic	Mechanical	Idiopathic	Genetic conditions*
Hypothyroidism	Scleroderma	Abnormal anatomy	Lysosomal storage diseases
Growth hormone	Physical activity	Obesity	MPS**
Pseudohypoparathyroidism	Fibrolipomatous hamartoma	Sports (golf, weight lifting, and basketball)	ML**
	Intraneural perineurioma	Playing instruments	Hunter syndrome**
	Hemangioma of the median nerve		Hurler syndrome**
	Macrodactyly		Congenital bone and muscle anomalies
	Fibrolipomas		Syndrome of HNPP
			Down syndrome
			Hemophilia
			Schwartz-Jampel syndrome
			Primary familial carpal tunnel syndrome
			Albright hereditary osteodystrophy
			Acromicric dysplasia
			Multiple xanthomas associated with familial hypercholesterolemia
			Klippel-Trénaunay syndrome
			Congenital constriction band syndrome

In future cases with children showing signs of ulnar neuropathy, we recommend more specific inquiries about activity level, including participation in throwing sports like track and field, baseball, softball, and football, frequent arm flexion, such as when they are eating or studying, bike riding, using a wheelchair, or knowledge about HNPP. Both syndromes should also be evaluated whenever there is suspicion of standalone carpal or cubital tunnel syndrome [8]. The Boston Carpal Tunnel Questionnaire, a reliable, frequently utilized tool for evaluating carpal tunnel syndrome, could be incorporated into clinical practice where patients are suspected of having carpal tunnel syndrome [9]. The Boston Carpal Tunnel Questionnaire uses a 55-point scale to look at symptom severity and functional status through 19 questions and was found to have good evidence of validity and reliability in a systematic review [10]. Patients should be screened for the carpal tunnel risk factors shown in Table 2 at their clinic visits [5,6,11]. It is also important to inquire about the classic symptoms of peripheral upper extremity neuropathies: numbness, tingling, wrist and elbow pain, and nighttime awakenings.

In cubital tunnel syndrome, clinical physical exam assessment should include Tinel’s test, elbow flexion compression test, and inquiry for signs of ulnar nerve subluxation such as “snapping” or “popping” sensations in the elbow [12]. Carpal tunnel syndrome manifests clinically with different physical signs and symptoms in the pediatric population. When assessing patients for carpal tunnel syndrome, physicians should evaluate the thenar muscles for weakness and atrophy, hand dexterity, and pain [5-7,11]. The classic median nerve neuropathy tests, including Tinel’s, Durkans, and Phalen’s tests, should be utilized [5-7,11]. In both neuropathies, EMG or nerve conduction studies are underutilized in the pediatric population due to the lack of parental consent, diagnostic accuracy, and the invasive nature of the procedure [5,6,10,12]. Previous studies of pediatric carpal tunnel syndrome suggest that ultrasound of the median nerve could be more effective for diagnosing carpal tunnel syndrome [6,7,12]. Due to children having a small, complex anatomy and decreased pain tolerance, ultrasound is a viable first-line diagnostic option to identify median neuropathy in children [6,7,11]. Nerve conduction studies are the gold standard for diagnosing peripheral neuropathies in adults and can still be utilized before and after surgical interventions in children. However, ultrasound is a safe and viable preoperative diagnostic tool that is particularly useful given that physical manifestations in children are easy to miss.

Previous studies demonstrate that operative treatment of adolescents and children is more effective than conservative management of ulnar and median nerve neuropathies [4]. In both neuropathies, surgical management has led to high levels of patient satisfaction [12]. Despite this, one review of compressive neuropathies in children found a failure rate of 23% among patients with standalone ulnar nerve compression in the cubital tunnel [6]. Another study exploring the outcomes of operative management of carpal tunnel syndrome in the pediatric population showed that only 50% of patients experienced full resolution of symptoms. In contrast, the remaining patients had only partial relief of symptoms [6,11]. In our case, the severity of the nerve damage was not clear from the physical exam and EMG studies. Still, intraoperatively, it was clear that the patient had significant neuropathy that could cause permanent dysfunction of the ulnar and median nerves. Based on our case and the current literature, we believe that including specific questions about family history and activity level will allow for more timely surgical intervention and improved outcomes for this pediatric patient population. As with adults, the pediatric population can utilize bracing, cortisone shots, and surgical management for carpal tunnel syndrome. However, none have been established as first-line treatments, regardless of the etiology [5-7,11]. Initial conservative management is recommended, but surgical management should be explored urgently to attempt to recover children fully. 

The rarity of entrapment neuropathies in the pediatric population prompted us to examine the rates of these conditions and their treatment in the TriNetX database (TriNetX, Inc., Cambridge, Massachusetts, United States) [13]. TriNetX is an online, global, federated health research database that provides access to electronic medical records from large healthcare organizations. TriNetX uses aggregated counts and statistical summaries of de-identified information, so no protected health information or personal data is accessible to users. The database was queried for pediatric patients who underwent carpal tunnel release, cubital tunnel release, or peroneal nerve release. The number of pediatric patients diagnosed with carpal and cubital tunnel syndrome in childhood was also determined. All queries and analyses were performed on 2023-09-15. The rates and demographic data are presented in Table 3. There were 20,819,207 pediatric patients on the network. Of these, 1,055 (0.005%) had undergone carpal tunnel release, 2,775 (0.01%) had undergone cubital tunnel release, 1,115 (0.005%) had undergone peroneal nerve release, and 503 (0.002%) were diagnosed with both carpal and cubital tunnel syndrome. The index event was defined as the release procedure for the first three cohorts and the date that carpal and cubital tunnel syndrome had been diagnosed for the fourth cohort. The average age at index ranged from 12 to 15.4 years. Most patients who underwent a release procedure were male, while most patients with carpal and cubital tunnel syndrome were female.

**Table 3:** Rates and demographics of pediatric patients with entrapment neuropathies on TriNetX (TriNetX, Inc., Cambridge, Massachusetts, United States)

Cohort	Number of patients	Rate % [(no./20,819,207) × 100]	Age at the index event (years, mean ± SD)	Sex (% M)
Carpal tunnel release	1,055	0.01%	12 ± 5.5	62%
Cubital tunnel release	2,775	0.01%	13.5 ± 4.7	58%
Peroneal nerve release	1,115	0.01%	12.4 ± 5.3	53%
Carpal + cubital tunnel syndrome	503	0.00%	15.4 ± 3.1	38%

The results of our TriNetX queries offer further insight into entrapment neuropathies in the pediatric population. The cohort of patients diagnosed with carpal and cubital tunnel syndrome was the smallest, which aligns with our inability to find any other reported cases of combined neuropathy in a pediatric patient. Nonetheless, 503 patients were diagnosed with both neuropathies, highlighting a potential lack of reporting in these cases. The sex differences observed were slight and likely coincidental, given the small number of patients. Our findings emphasize the rarity of entrapment neuropathies in children and highlight the need for further study of these cases.

## Conclusions

Based on our case and the current literature, we believe that incorporating specific questions about family history and activity level can lead to more timely surgical intervention and improved outcomes for pediatric patients. Additionally, it is crucial to simultaneously evaluate both neuropathies in children. In our case, the severity of nerve damage was unclear from the physical exam and EMG studies, which may have contributed to a delay in surgical intervention and more severe nerve damage. Although our patient recovered well from surgery, she continued to experience persistent pain, paresthesias, and weakness. Consequently, she is taking gabapentin for symptom management and is following up with neurology.
